# Intussusception in an adult with a history of rectal cancer: a case report

**DOI:** 10.3389/fmed.2025.1561289

**Published:** 2025-05-15

**Authors:** Wei Liu, Feng Li, Cheng Jiao, Jun Guo, Yurong Jiao, Guangchao Liu, Yao Zhang

**Affiliations:** Department of General Surgery, Bethune International Peace Hospital, Shijiazhuang, Hebei, China

**Keywords:** adult intussusception, rectal cancer, surgical treatment, CT scan, case report

## Abstract

This case report details the clinical journey of a 37-year-old male patient who had undergone rectal cancer surgery five years prior to symptom onset. The patient presented with an abdominal mass and intermittent abdominal pain that had been present for approximately two weeks prior to the current hospitalization for treatment. Through a comprehensive array of diagnostic procedures, notably abdominal CT scans and colonoscopies, the presence and precise location of intussusception were ascertained. The surgical management entailed a radical right hemicolectomy supplemented by preventive measures against recurrence. In the postoperative phase, the patient was administered oral medications and subjected to regular follow-up. This case highlights the diagnostic and therapeutic challenges of adult intussusception in postoperative cancer patients and underscores the importance of a multidisciplinary approach. It also emphasizes the need for tailored treatment strategies to optimize patient outcomes.

## 1 Introduction

Intussusception, first documented in 1674 and subsequently detailed as “intussusception” in 1789, is characterized by the telescoping of one segment of the intestine and its mesentery into an adjacent segment, resulting in acute intestinal obstruction (1). This condition can cause luminal obstruction and compromised blood flow (2). If diagnosis and intervention are delayed, complications such as intestinal ischemia, necrosis, and perforation can occur, potentially leading to increased mortality (3). While more prevalent in children, particularly infants aged 5 to 9 months (4), intussusception is relatively rare in adults, particularly in patients with a past history of malignancy (5, 6). In adults, the presence of a pathological lead point is a common cause, observed in approximately 80% of cases (7).

For patients who have previously undergone rectal cancer surgery, the development of intussusception presents unique diagnostic and therapeutic challenges. Anatomical and physiological alterations resulting from the prior surgical intervention can complicate clinical manifestations and the interpretation of diagnostic tests. Moreover, the potential for cancer recurrence or postoperative complications further amplifies the complexity of managing such cases. A multidisciplinary approach, involving collaboration among surgeons, radiologists, oncologists, and other specialists, is crucial for addressing these challenges. This case report aims to present a detailed account of the diagnosis and treatment process of a 37-year-old male patient who experienced intussusception five years after rectal cancer surgery. By sharing this experience, we hope to expand the existing knowledge base and provide valuable insights for clinicians who may encounter similar situations in their practice.

## 2 Case description

The patient, a 37-year-old male, presented with an abdominal mass and intermittent abdominal pain that had presented for approximately two weeks before hospitalization. Initially, the pain was mild but progressively worsened. He also experienced abdominal distension, which lead to a decrease in food intake. His bowel movements continued, primarily consisting of loose stools, occasionally black. Importantly, he had no recent history of trauma or gastrointestinal infection. The initial evaluation involved a multidisciplinary team, including surgeons, radiologists, and gastroenterologists, to comprehensively assess the patient’s clinical symptoms and diagnostic findings. Upon physical examination, the patient appeared cachectic, with a BMI of 17.72. Abdominal palpation revealed a firm mass, approximately 10 cm in diameter, located in the right lower quadrant, which was tender but without rebound tenderness. The remainder of the abdominal examination, including rectal examination, yielded no significant findings. Laboratory investigations indicated a hemoglobin level of 76 g/L, consistent with anemia. White blood cell and platelet counts were within normal limits. Notably, the stool occult blood test was positive. Biochemical assays revealed slightly decreased potassium and sodium levels. However, tumor markers, such as CEA, were within normal limits.

In 2018, the patient had radical rectal cancer surgery at an external institution. The postoperative pathology showed a 6.5 cm × 5.5 cm × 1 cm ulcerated mass, diagnosed as grade II adenocarcinoma with mucinous components, infiltrating peristomal adipose tissue. Lymph node metastasis: paracolon 1/27, mesentery 0/8, mesenteric root 0/4, group 253 0/4. Immunohistochemistry (IHC): BRAF (−), MLH1 (+), MSH2 (+), MSH6 (+), PMS2 (−). After that, he received the XELOX chemotherapy regimen.

**FIGURE 1:**
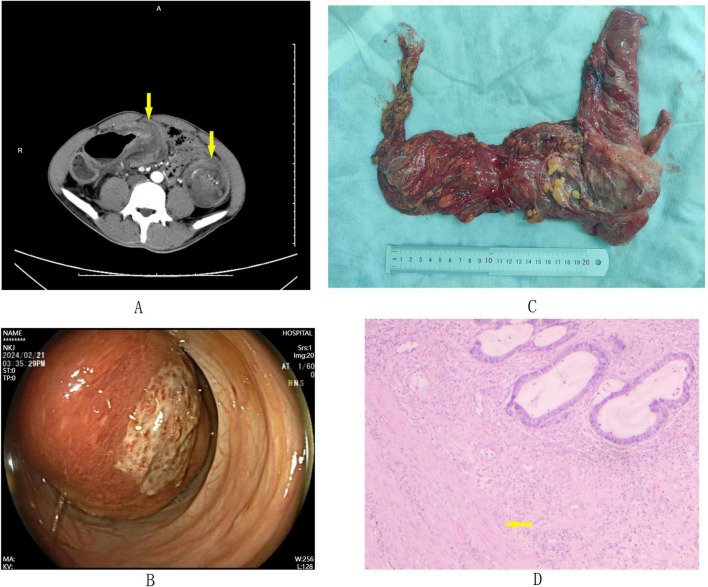
Integrated figure panel. **(A)** Abdominal CT scan: Axial images showing the transverse colon intussusception with the characteristic “banana sign” and the descending colon intussusception with the “target sign” (yellow arrow). **(B)** Enteroscopic examination: Image showing significant mucosal bulging at the site of intussusception. **(C)** Surgical Specimen: The specimen includes the distal ileum, cecum, ascending colon, and right half of the transverse colon. **(D)** Pathological Finding: Cross-sectional view of the surgical specimen (×10), highlighting the adenocarcinoma invading the muscularis propria (yellow arrow).

CT scans performed outside the hospital revealed two intussusceptions, located in the transverse and descending colon (Figure 1A). However, upon hospitalization and repeat CT scans, intussusception was still evident in the transverse colon, while none was detected in the descending colon. Enteroscopic examination revealed anastomotic scarring 7 cm from the anus and significant mucosal bulging 37 cm away, obstructing the intestinal lumen with a congested, erosive surface, which hindered further scope advancement (Figure 1B). During the current surgical intervention, it was found that from the ileocecal part to the middle of the transverse colon, the intestinal tubes were tortuously intussuscepted, about 15 cm long. The bowel wall was severely edematous. The patient’s colonic mesentery was approximately three times longer than that of a normal adult. After reducing the intussusception, a bulging mass was seen at the ileocecal part, suspected to be malignant. With the consent of the patient’s family, a radical right hemicolectomy was carried out (Figure 1C). Further exploration of the descending colon showed no obvious pathological changes. Considering the patient’s history of descending colonic intussusception and the elongated colonic mesentery increasing recurrence risk, the descending colon was fixed to the left side of the peritoneum to prevent recurrence.

Postoperatively, the patient’s vital signs were stable, and he recovered well from the surgical stress. Pathologic analysis of the specimen showed a 5 cm × 5 cm × 3 cm ileocecal mass, diagnosed as grade II adenocarcinoma with some mucinous adenocarcinoma, invading extramuscular fibro-fatty tissue (Figure 1D). Lymph node metastases were 6/18 in paracolon, 0/12 in mesentery, 0/10 in mesenteric root, with cancerous infiltration at appendiceal orifice but no cancer in appendix. The tumor was staged as pT3N2aM0 IIIB according to the TNM classification system. IHC results were HER-2 (2+), not amplified by FISH, MLH1 (+), MSH2 (+), MSH6 (+), PMS2 (+), P53 (+, 10%), Ki-67 (+, 70–80).

Following the current surgical intervention, given the patient’s financial constraints and refusal of the standard chemotherapy regimen, the oncologists and medical team collaborated to devise an alternative treatment plan involving oral chemotherapy (capecitabine) and antiangiogenic therapy (apatinib). The medications and dosages were meticulously adjusted according to his condition. Regular follow-up visits were scheduled every 3 months to monitor his recovery and identify any potential relapses or complications. As of the last follow-up, the patient has been followed up for a total of 12 months postoperatively without evidence of recurrence or significant complications.

For a detailed timeline of the patient’s clinical journey, including key events and dates, please refer to Table 1. Additionally, supplementary imaging and histopathological findings are provided in Supplementary Figures 1–3.

**TABLE 1:** Timeline with relevant data.

Date	Event
2018.06.21	Patient had radical rectal cancer surgery at an external institution.
2024.01.26	Patient presented with an abdominal mass and intermittent abdominal pain.
2024.02.03	Out-of-hospital CT scans revealed two intussusceptions, located in the transverse and descending colon.
2024.02.18	Hospitalization for repeat CT scans, intussusception still evident in the transverse colon, none detected in the descending colon. Enteroscopic examination revealed anastomotic scarring and significant mucosal bulging.
2024.02.24	Surgical intervention, radical right hemicolectomy performed.
2024.03.01	Postoperative pathological analysis showed a 5 cm × 5 cm × 3 cm ileocecal mass, diagnosed as stage IIIB adenocarcinoma.
2024.03.20	Patient refused the initially recommended chemotherapy regimen, treatment with capecitabine and apatinib initiated.

## 3 Discussion

Adult intussusception is a rare condition in clinical practice, accounting for approximately 1% of adult intestinal obstructions and less than 5% of all intussusceptions (8). Unlike pediatric intussusception, over 90% of adult cases are attributed to organic pathology (9). Small bowel-colon (ileocecal) intussusception is more prevalent, with the majority of these being malignant tumors (7). Furthermore, intussusception can be triggered by benign tumors, polyps, postoperative adhesions, Meckel’s diverticulum, and Peutz-Jeghers syndrome (10). Abdominal pain is the most commonly reported symptom, affecting about 86.23% of patients. It is often accompanied by nausea, vomiting, diarrhea, hematochezia, abdominal mass, and distention (11). In this case, the patient’s history of rectal cancer surgery and non-specific symptoms such as intermittent abdominal pain, abdominal distension, and changes in bowel habits initially suggested a potential recurrence of rectal cancer, complicating the diagnostic process.

Abdominal CT is a highly sensitive diagnostic tool, with an accuracy rate of 77.8% (12). It can show the location, extent, and morphology of intussusception, and assess the mesenteric vasculature, providing key diagnostic details. Contrast-enhanced CT can help distinguish between benign and malignant tumors, although it may have limitations in identifying the nature of the lead point in some cases (13). Abdominal ultrasound, being non-invasive, radiation-free, and easy to perform, has a diagnostic accuracy of about 49.2% for adult intussusception (12). However, its effectiveness can be compromised by factors such as obesity and intestinal gas. Additionally, barium enema and fiberoptic colonoscopy offer diagnostic insights in select cases. In this patient, combining CT scans and colonoscopy was essential for developing an appropriate treatment plan. Notably, the transition from an initial diagnosis of two colonic intussusceptions to a confirmed diagnosis in the transverse colon was followed by spontaneous resolution of the intussusception in the descending colon. This phenomenon may be related to retrograde intestinal peristalsis, thereby highlighting the complexity of this case.

Regarding treatment strategies, adult intussusception typically requires surgical intervention, unlike pediatric intussusception. For the majority of adult patients, especially those with colonic intussusception, bowel segment resection and anastomosis are preferred over reduction due to the high incidence of malignant tumors (11). This approach minimizes the risks associated with tumor cell dissemination within the bowel and progression to venous metastasis. During the intraoperative phase, the choice between open and laparoscopic surgery depends on various factors, including the location of the intussusception, its underlying cause, and the patient’s overall physical condition (6). Laparoscopic surgery, known for its reduced trauma and faster postoperative recovery, can be effectively applied in certain cases. However, for patients presenting with acute intestinal obstruction, significant bowel dilation, or complex pathological conditions, open surgery remains the more suitable option. In this particular patient, given his prior abdominal surgery that likely led to alterations in the intestinal tract’s structure and function, coupled with symptoms indicative of intestinal obstruction, open surgical intervention was deemed necessary. Intraoperatively, a mass was detected in the ileocecal region, which had induced intussusception extending as far as the transverse colon. In accordance with established oncological principles, a radical right hemicolectomy was performed. Additionally, considering the patient’s history of descending colonic intussusception and mesenteric anomalies, the descending colon was fixed to prevent potential recurrence, thereby emphasizing the individualized nature of the surgical treatment approach.

Multiple Primary Colorectal Cancer (MPCRC) is clinically defined as the presence of at least two distinct colorectal cancer tumors, classified as Synchronous Colorectal Cancer (SCRC) and Metachronous Colorectal Cancer (MCRC) (14). MPCRCs account for 5%–10% of all colorectal cancers (15). Some MPCRC are associated with genetic patterns, such as Lynch syndrome (LS), familial adenomatous polyposis (FAP), and serrated polyp syndrome, which increase the risk of MPCRC. There appears to be a familial pattern in MCRC, while individual factors are more important in SCRC. Environmental factors such as smoking, cooking food at high temperatures, and alcohol consumption may increase the risk of MPCRC. Chromosomal instability, microsatellite instability and gene methylation are involved in SCRC and MCRC (16). Different clinical features may suggest individual susceptibility to MPCRC, such as age, gender, and tumor location. The implementation of risk-stratified management algorithms, incorporating oncologically radical resections and molecularly-informed surveillance protocols, has shown potential to optimize therapeutic outcomes in MPCRC (17). Initial IHC evaluation of the rectal cancer specimen showed loss of PMS2 expression, with retention of MLH1, MSH2, and MSH6, suggesting a partial mismatch repair (MMR) deficiency. This pattern is atypical for Lynch syndrome but cannot be ruled out. Subsequent analysis of the ileocecal adenocarcinoma demonstrated preserved expression of all four MMR proteins (MLH1, MSH2, MSH6, and PMS2), indicating a sporadic rather than hereditary origin.

This case reveals a stage III B adenocarcinoma of the ileocecum, characterized as pT3N2aM0 according to the TNM classification system. Given the patient’s medical history, it is categorized as MCRC. Although the patient achieved relatively stable short-term postoperative recovery, the long-term risk of tumor progression remains considerably high. It is crucial to formulate a standardized adjuvant treatment protocol and implement rigorous follow-up surveillance. In this case, financial limitations prevented the patient from affording genetic susceptibility testing, and he declined the standard chemotherapy regimen. In response, our medical team opted for oral chemotherapy combined with antiangiogenic therapy, along with close follow-up monitoring. This tailored approach aims to balance the patient’s treatment needs with his financial constraints while striving to achieve the best possible therapeutic outcome.

## 4 Conclusion

This case highlights the diagnostic and therapeutic challenges of adult intussusception, particularly in a patient with a history of rectal cancer. Accurate diagnosis required the integration of clinical manifestations, imaging studies, and supplementary diagnostic data. The treatment plan was customized to the patient’s unique circumstances, underscoring the importance of individualized care. As a single case report, the findings may not be generalizable to a broader patient population. Further research is warranted to refine diagnostic and therapeutic strategies for adult intussusception, particularly in cancer patients, to enhance long-term outcomes.

## Data Availability

The raw data supporting the conclusions of this article will be made available by the authors, without undue reservation.
