# Supplementary material for: Intussusception in an adult with a history of rectal cancer: a case report
Source: Front Med (Lausanne). 2025 May 15;12:1561289. doi: 10.3389/fmed.2025.1561289 (PMC12119493; doi:10.3389/fmed.2025.1561289)
Supplement: Supplementary file 1 [file Data_Sheet_1.docx]

**Table S1. Previous Rectal Anastomosis (metal anastomosis line).**

| 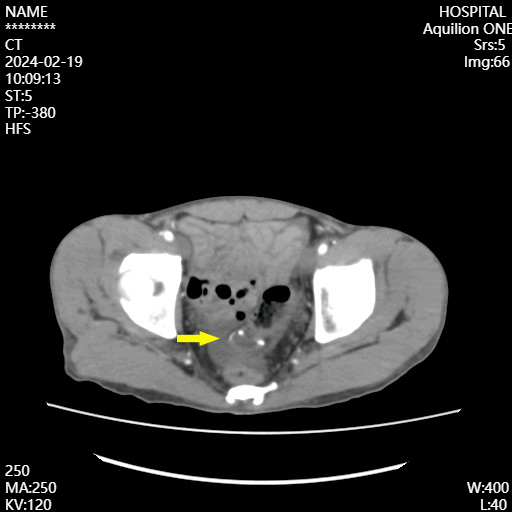 | 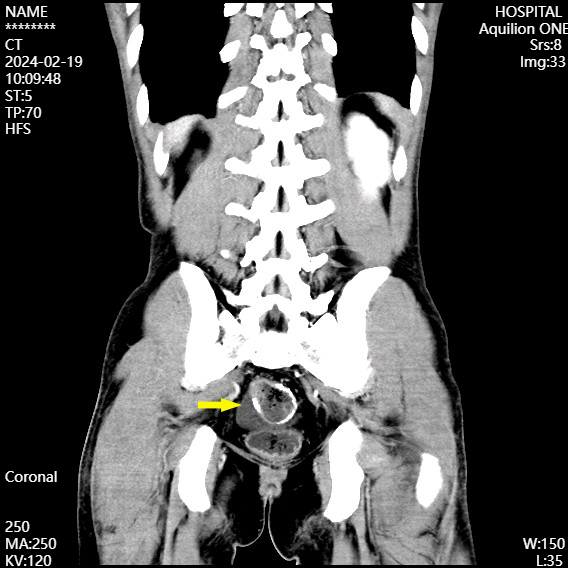 |
| --- | --- |
| Cross section (yellow arrow) | Coronal plane (yellow arrow) |

**Figure S2. Histopathological and Immunohistochemical Findings**

| 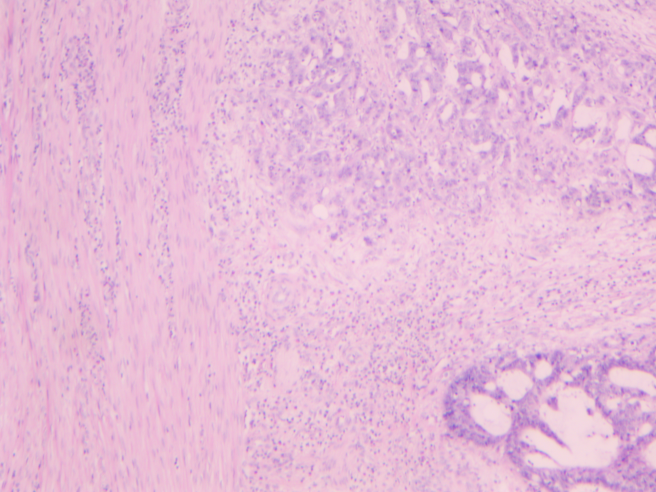 | 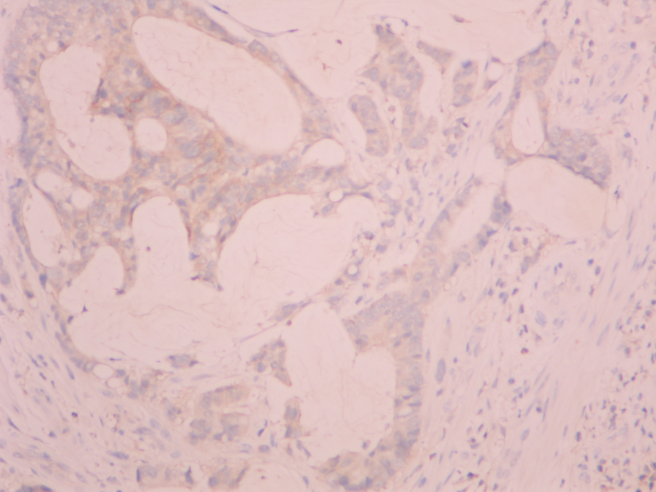 |
| --- | --- |
| H&E staining of the surgical specimen | HER2: Over expression (2+) |
| 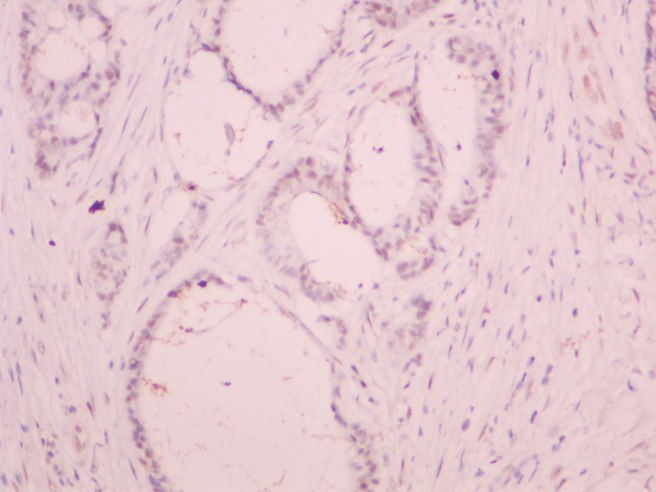 | 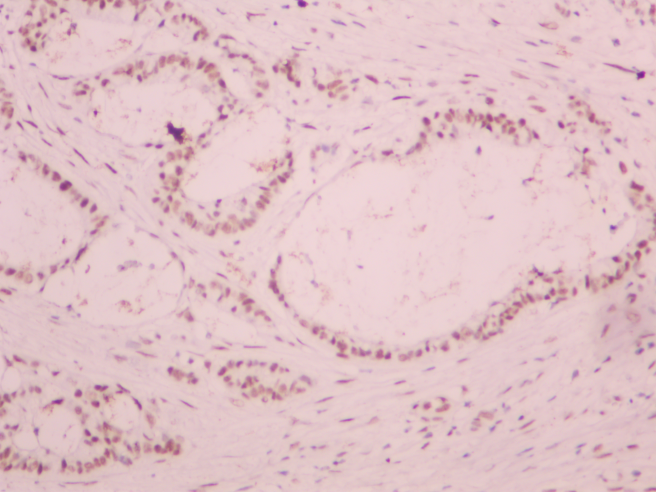 |
| MLH1: Positive | MSH2: Positive |
| 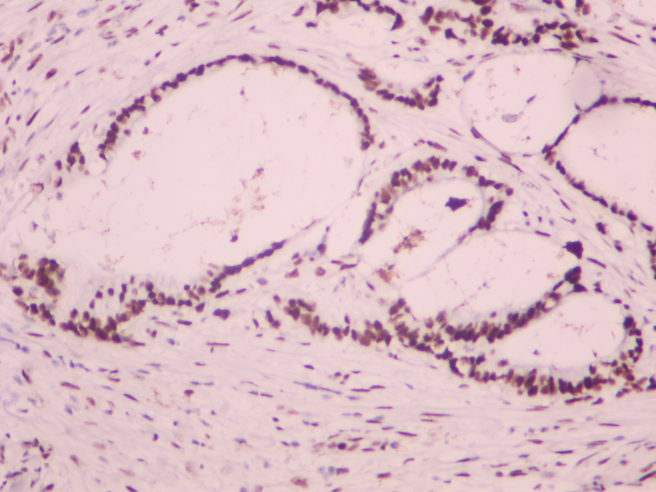 | 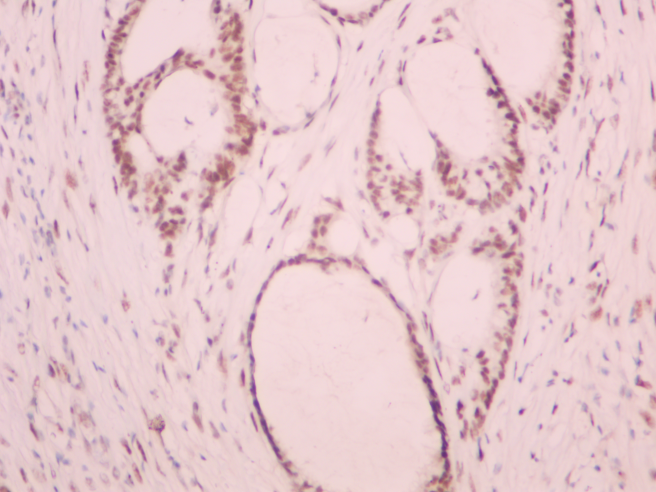 |
| MSH6: Positive | PMS2: Positive |
| 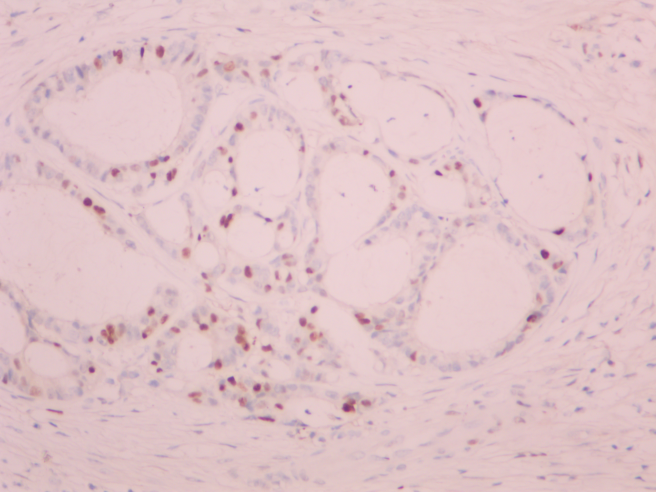 | 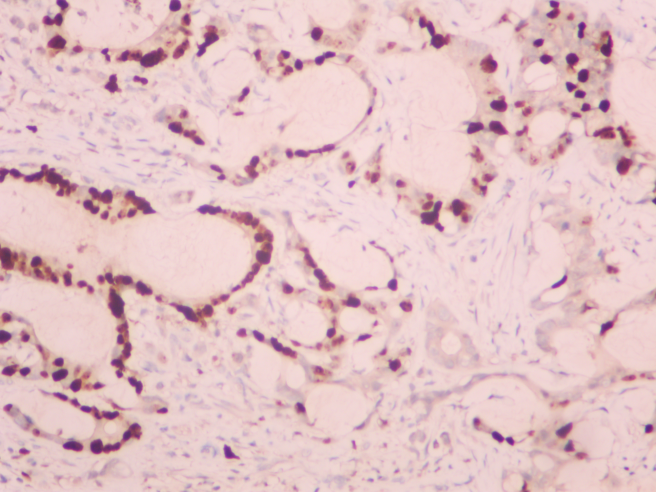 |
| P53: Positive in 10% of cells | Ki-67: Positive in 70-80% of cells |

**Figure S3. Timeline with Relevant Data**

**
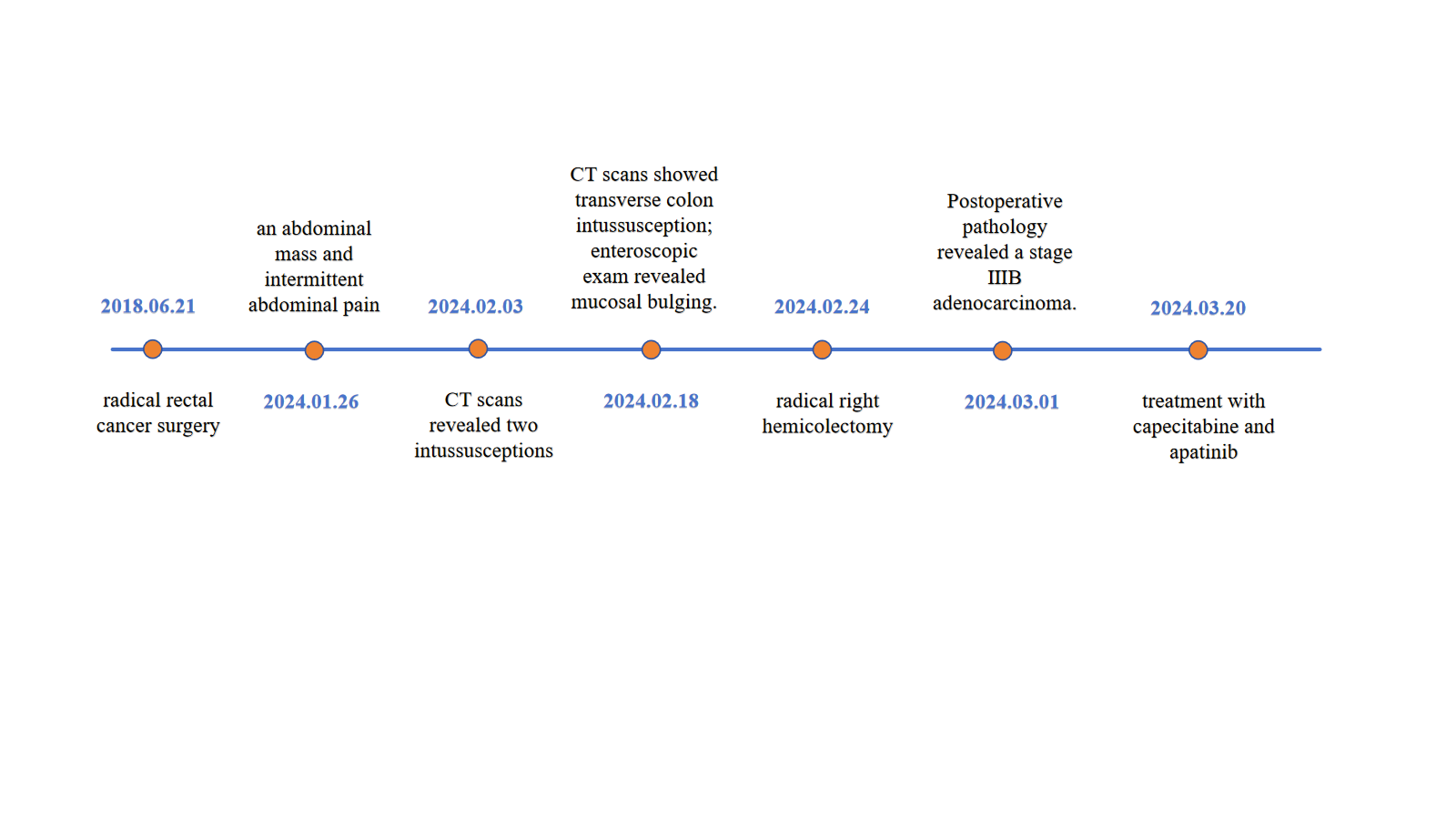
**
